# Effects of Shared Word Order on Intrasentential Language Mixing in English-Dutch, Polish-Dutch, and Turkish-Dutch Bilingual Children

**DOI:** 10.3390/bs16060839

**Published:** 2026-05-22

**Authors:** Vera Snijders, Ora Oudgenoeg-Paz, Merel van Witteloostuijn, Elma Blom

**Affiliations:** Department of Education and Pedagogy, Faculty of Social and Behavioural Sciences, Utrecht University, Heidelberglaan 1, 3584 CS Utrecht, The Netherlands; o.oudgenoeg@uu.nl (O.O.-P.); m.t.g.vanwitteloostuijn@uu.nl (M.v.W.); w.b.t.blom@uu.nl (E.B.)

**Keywords:** language mixing, code-switching, bilingualism, mixed sentence repetition, word order overlap

## Abstract

Bilingual children commonly mix languages. Their language mixing generally adheres to grammatical constraints, yet it may impose processing and production costs. This study examined how 4-to-7-year-old English-, Polish-, and Turkish-Dutch bilingual children processed and repeated mixed-language sentences. It aimed to (a) determine whether they struggle with mixed-language sentences, (b) study whether shared word order in either the main or subordinate clause facilitates repetition, (c) compare the effects of different types of mixing, i.e., insertion and alternation, and (d) link error rates to daily mixing experience. Fifty-seven children participated in a mixed sentence repetition task. Mixed Dutch sentences with embedded elements from other languages in the task enable the examination of the role of clause and mixing type across four types of sentences: (1) main clause insertion, (2) subordinate clause insertion, (3) main clause alternation, and (4) subordinate clause alternation. In addition, monolingual Dutch sentences with main and subordinate clauses allow investigation of the effects of processing mixed sentences. The results of the generalized linear mixed-effects models with error rates as the outcome variable suggest that mixing may play a limited role. We also found no evidence of a relation between task performance and daily mixing experience. These results provide no support for processing and production costs associated with language mixing. We discuss these results in light of theories on language mixing, previous research and methodological considerations.

## 1. Introduction

Mixing languages is a common and natural part of bilingual children’s language development. Yet, language mixing sometimes causes concern among parents and practitioners, who may be worried that children confuse their languages or experience a problematic language development. From previous research, however, we know that when bilinguals mix their languages, they take into account the grammatical constraints of both languages (Meisel, 1994), suggesting that language mixing is not the result of confusion, but rather based on knowledge of grammatical constraints. At the same time, balancing the grammatical constraints of both languages may be effortful for processing (Gollan & Goldrick, 2016) and producing mixed language (Myers-Scotton & Jake, 2009). Grammatical constraints may be associated with different sentence properties. For example, if word order overlaps between languages, mixing is facilitated (Deuchar, 2005; Poplack, 1980). Considering word order may be particularly relevant in case mixing involves grammatical aspects instead of being limited to single lexical items (Muysken, 2000). In this study, we aim to contribute to our understanding of these processing and production costs associated with children’s language mixing. We do this based on several sentence properties, i.e., monolingual vs. bilingual, shared word order vs. non-shared word order, and insertion vs. alternation, using an experimental mixed sentence repetition task in English-Dutch, Polish-Dutch, and Turkish-Dutch bilingual children. Additionally, we address the ecological validity of this experimental task to assess language mixing (Blanco-Elorrieta & Pylkkänen, 2017; Gross et al., 2022).

### 1.1. Language Mixing Across Grammatical Systems

Language mixing refers to the use of multiple languages within a conversation and can occur between sentences (i.e., intersentential mixing) or within sentences (i.e., intrasentential mixing; Meisel, 1994). The present study investigates intrasentential mixing, which has also been the focus of several theoretical models on language mixing or code-switching^1^. Perhaps the most well-known is the Matrix Language Frame (MLF) model (Myers-Scotton, 1993), which states that when language mixing occurs, one language (i.e., the matrix language) supplies the morphosyntactic frame, and lexical items from the other language (i.e., the embedded language) are slotted into this frame. The grammaticality of the sentence is then based on consistency with the grammatical rules of the matrix language. Other models take into account the grammatical systems of both languages; for example, the Equivalence Constraint (Poplack, 1980) poses that mixing only happens at points where the syntactic rules of both languages overlap, thereby preventing mixing at points where syntactic rules are incongruent. Focusing on bilingual children, Meisel (1994) proposes that mixing is only possible when it does not violate the rules of both grammatical systems that the children draw on. Together, these theoretical models highlight that language mixing is subject to grammatical constraints, and that mixing where grammatical systems do not overlap is unlikely.

However, empirical evidence for the role of grammatical overlap in naturalistic data is inconsistent. On the one hand, some studies demonstrate that children indeed take into account grammatical constraints when mixing their languages. For example, a relatively large case study of 15 children showed that, when mixing French and English, children obey the constraints set by the MLF model the majority of the time (Paradis et al., 2000). Another study of nine children demonstrated that children mix between English and Cantonese when grammatical rules regarding word order and verb usage overlap (Yip & Matthews, 2016). Other studies have, however, found various instances where children mix structures that do not overlap between grammatical systems (e.g., Vihman, 2018; for reviews, see Treffers-Daller, 2022; Vihman & Vihman, 2026), highlighting that findings regarding the role of grammatical constraints are mixed. Moreover, much of the work on bilingual children’s language mixing is based on naturalistic case studies. Naturalistic case studies can provide in-depth insights into children’s language mixing behavior but are less suited for a systematic investigation into the role of grammatical overlap, because these case studies are difficult to generalize to larger populations due to the small sample size and lack of control over the research setting. A useful way to study the effect of overlap is through experimental methods, such as sentence repetition tasks (Gullberg et al., 2009), to allow manipulation of word order, as well as other sentence properties.

### 1.2. Mixed Sentence Repetition Task

In sentence repetition tasks, participants hear sentences which they have to repeat. Sufficiently long sentences force children to rely on grammar, not working memory, to repeat them (Klem et al., 2015). Consequently, errors in repetition may reveal gaps in children’s grammatical knowledge. Sentence repetition is often applied to measure such grammatical knowledge in both research (Kałowski et al., 2025) and clinical settings (Rujas et al., 2021). In mixed sentence repetition, participants repeat sentences that contain a switch from one language to another (Azuma & Meier, 1997; Clyne, 1972; Hofweber & Marinis, 2023; Lipski, 2017; Soesman & Walters, 2021; Soesman et al., 2022). The following sections detail how sentence properties may impact children’s performance on a mixed sentence repetition task, focusing on the specific sentence properties investigated in the current study, namely monolingual vs. bilingual sentences, word order overlap in mixed sentences (i.e., shared word order vs. non-shared word order) and mixing type (i.e., insertion vs. alternation).

#### 1.2.1. Monolingual vs. Bilingual Sentences

First, when compared with monolingual sentence repetition, mixed sentence repetition tasks can be used to test whether repeating mixed sentences is relatively effortful. Bilinguals activate multiple languages simultaneously (Colomé, 2001; Hermans et al., 1998) and often must select the appropriate language while inhibiting the other language (Green & Wei, 2014). When switching to the other language, bilinguals need to overcome this inhibition, resulting in additional processing and production costs (Green & Abutalebi, 2013). In the context of a mixed sentence repetition task, mixed sentences may require additional processing efforts compared to monolingual sentences, as bilinguals have to take into account (the grammatical rules of) two languages (Gollan & Goldrick, 2016). It is also possible that additional production efforts are needed, since the production of structurally well-formed mixed sentences may require greater effort in terms of sentence construction and congruency checking (Myers-Scotton & Jake, 2009). Research on language mixing has demonstrated increased costs associated with switching between languages across a variety of methods (for reviews, see Declerck, 2020; Declerck & Philipp, 2015). For example, research on mixed sentence processing has indeed shown that children require additional processing time when presented with mixed-language compared to single-language stimuli (Byers-Heinlein et al., 2017; Gross et al., 2019). Increased costs have also been found in mixed sentence repetition tasks. In adults, repeating sentences that contained mixing between German and English (Hofweber & Marinis, 2023) or between Quichua and Ecuadorian Media Lengua (Lipski, 2017) was more costly than repeating monolingual sentences as measured by accuracy. In 5;5-to-6;5-year-old bilingual children, repeating sentences that contained mixing in different components of prepositional phrases (PPs) also resulted in lower accuracy than repeating monolingual sentences in English and Hebrew (Soesman & Walters, 2021). Although the costs associated with repeating mixed sentences has been shown, studies specifically targeting children remain limited, highlighting the need for further research.

#### 1.2.2. Word Order Overlap

Second, comparisons of different language combinations as well as different sentence structures can be used to test whether and how word order overlap affects children’s repetition performance. Mixed sentence repetition allows us to manipulate sentences so that mixing occurs where word orders between languages do not overlap, potentially resulting in additional costs due to incongruent syntactic structures (Meisel, 1994; Myers-Scotton, 1993; Poplack, 1980). Repeating such sentences may result in higher error rates compared to repeating mixed sentences that adhere to the grammatical rules imposed by both languages. Previous research on mixed sentence repetition has focused on the accuracy with which bilinguals repeat mixed sentences. These studies have shown that it is indeed harder to repeat sentences that require increased grammatical integration or that mix non-overlapping structures. Adult studies have shown increased difficulties with repeating sentences that contain (a) dense code-switching (Hofweber & Marinis, 2023); (b) within-clause mixing compared to mixing at the clause boundary (Clyne, 1972); (c) and function words compared to content words (Azuma & Meier, 1997; Lipski, 2017). So far, only two studies have used a mixed sentence repetition task to study the role of non-overlapping grammatical structures in language mixing in children. The first study showed that varying processing costs were associated with mixing of different components within PPs between English and Hebrew in 5;5-to-6;5-year-old bilingual children (Soesman & Walters, 2021). Sentences in which the mixed component(s) of the PPs were incongruent with the grammatical rules of both languages (e.g., switch points at prepositions or determiners) resulted in lower accuracy than sentences in which there was congruency between the two grammatical systems (e.g., switch points at nouns or full PPs). Children were also more likely to demonstrate non-targeted mixing (i.e., mixing other than what was included in the task) in mixed compared to monolingual sentences (Soesman et al., 2022), especially in sentences with more grammatical constraint violations. These studies indicate that children are more likely to experience difficulty in the case of non-overlapping grammatical structures. However, research in children remains limited to PPs, and therefore, it remains to be seen whether similar results are found when mixing other word order differences.

#### 1.2.3. Mixing Type: Insertion vs. Alternation

Third, we explore different types of intrasentential mixing (i.e., insertion and alternation; Muysken, 2000). Insertion concerns the embedding of lexical elements into the structure of another language, illustrated in example (a) below. It does not require the speaker to juggle two grammatical systems at once and can thus be done with minimal costs (Muysken, 2000). Alternation, on the other hand, comprises grammatical elements, as illustrated in example (b) below. It requires more grammatical integration between two languages than insertions and is potentially more costly to process and produce (Muysken, 2000).

- Het meisje proeft de ORANGE.
  The girl tastes the orange.
- Het meisje TASTES THE ORANGE.
  The girl tastes the orange.

Hardly any research has investigated mixing type using mixed sentence repetition. One study found that adults make more errors repeating sentences containing insertions compared to alternations, although this difference was not significant (Hofweber & Marinis, 2023). While this finding is not in line with Muysken’s (2000) prediction, this may be explained by the fact that participants in this study were L1-German L2-English adult bilinguals in an L2 context who did not encounter much insertional mixing in their daily lives (Hofweber & Marinis, 2023). The finding that insertions were slightly harder to repeat than alternations may therefore be a reflection of experience, rather than increased costs associated with mixing itself. To our knowledge, there is currently no research addressing these mixing types in children. This gap underscores the need for studying insertions and alternations in a sample of children, where such mixing may be more frequent.

### 1.3. The Current Study

In this study, we examine the processing and production costs associated with children’s language mixing (Gollan & Goldrick, 2016; Myers-Scotton & Jake, 2009) and help further develop theories on how children mix their languages and how they apply grammatical constraints when doing so (Meisel, 1994; Myers-Scotton, 1993; Poplack, 1980). To this end, we use a mixed sentence repetition task in which English-Dutch, Polish-Dutch, and Turkish-Dutch bilingual children (age 4–7) repeat different types of sentences that contain mixed language in either the main or subordinate clause. The current study has four aims.

The first aim is to compare children’s ability to repeat monolingual and mixed sentences. Previous research suggests that the processing and production of mixed language is more costly than monolingual language (Gollan & Goldrick, 2016; Myers-Scotton & Jake, 2009; Soesman & Walters, 2021). Based on this research, we hypothesize the following:

**Hypothesis** **1.** *Children have less difficulty, and thus make fewer errors, repeating monolingual sentences compared to sentences that contain language mixing.*

The second aim is to examine whether bilingual children’s ability to repeat mixed sentences is facilitated by shared word order between Dutch and the minority language. We consider the relative positions of the object (O) and verb (V) in English, Polish, and Turkish that partially overlap with Dutch word order, though in different ways. Examples of the word order structure of these four languages can be found in Table 1a. Dutch main clauses comply with the Verb Second rule, which implies that the finite verb is in second sentence position (i.e., VO), exemplified in (i). In contrast, in Dutch subordinate clauses, the finite verb follows the object (OV) (Koster, 1975), as demonstrated in (ii). Regardless of clause type, Polish and English sentences follow the VO order, illustrated in (iii), while Turkish sentences have an OV order, shown in (iv). Mixing between non-overlapping structures in a mixed sentence repetition task may be more effortful than mixing between overlapping structures. Table 1b presents examples of mixing in the main (v) and subordinate (vi) clause, illustrating what congruent and incongruent language mixing between the grammatical structures of Dutch and the minority languages looks like. Based on theory (Meisel, 1994; Poplack, 1980) and previous research (Soesman & Walters, 2021), we propose the following hypotheses, which are also illustrated in Table 2:

**Hypothesis** **2.** *Polish-Dutch and English-Dutch children (SVO) have less difficulty and thus make fewer errors, when switching in a main clause compared to a subordinate clause.*

**Hypothesis** **3.** *Turkish-Dutch children (SOV) have less difficulty, and thus make fewer errors, when switching in a subordinate clause compared to a main clause.*

**Hypothesis** **4.** *In main clauses, Polish-Dutch and English-Dutch children (SVO) have less difficulty switching, and thus make fewer errors, than Turkish-Dutch children (SOV).*

**Hypothesis** **5.** *In subordinate clauses, Turkish-Dutch children (SOV) have less difficulty switching, and thus make fewer errors, than Polish-Dutch and English-Dutch children (SVO).*

The third aim is to explore the effects of mixing type (i.e., insertion and alternation) on bilingual children’s ability to repeat mixed sentences. Based on theoretical considerations (Muysken, 2000), we tentatively hypothesize the following:

**Hypothesis** **6.** *Children have less difficulty, and thus make fewer errors, repeating sentences that contain insertions compared to sentences that contain alternations.*

The fourth aim is to study the relation between bilingual children’s ability to repeat mixed sentences and their experience with intrasentential mixing in daily life. This mixed sentence repetition task, along with other experimental tasks that target language mixing, manipulates children’s language choice and use. Mixing in this task may not directly reflect their language mixing in daily life (Blanco-Elorrieta & Pylkkänen, 2017). Previous work has not addressed this relation through mixed sentence repetition in children, but one study reported that children with more mixing experience in daily life also mixed more in a scripted confederate dialogue task (Gross et al., 2022). Moreover, experience with specific mixing types was related to mixed sentence repetition performance in adults (Hofweber & Marinis, 2023). We hypothesize the following:

**Hypothesis** **7.** *Children have less difficulty, and thus make fewer errors, repeating sentences that contain intrasentential language mixing if they encounter more intrasentential mixing in daily life.*

## 2. Materials and Methods

### 2.1. Procedure

The current study is part of a larger research project (Children and Language Mixing: developmental, psycholinguistic and sociolinguistic aspects; CALM; https://osf.io/p9gje/). This research project has been approved by the Ethics Review Board of the Faculty of Social and Behavioral Sciences at Utrecht University (FETC 20-0291). The study was preregistered on the Open Science Framework (OSF; https://osf.io/96z8d preregistered on 4 September 2025).

The data were collected in the Netherlands from July 2022 to March 2025 in two waves. Children and their families were recruited via flyers distributed through churches, locally organized events, mosques, personal networks, schools, and social media. In each wave, data collection consisted of two home visits of approximately 2 h each. In addition to the measures reported here, other tests and questionnaires were administered, which are not relevant to the current study. Parents provided informed consent during the first home visit. During the first home visit, the researcher spoke English, Polish, or Turkish as well as Dutch. The mixed sentence repetition task, as well as vocabulary tests in English, Polish, or Turkish, were administered during this visit. During the second home visit, the researcher spoke Dutch only. Dutch vocabulary and grammar were assessed and parents filled in the Quantifying Bilingual Experience questionnaire (Q-BEx; de Cat et al., 2025) together with the researcher. The average time between two visits was around 3 weeks (*M* = 3.01, *SD* = 2.03 weeks). Parents received a compensation of 15 euros in cash per visit. Children received a diploma with stickers for each completed task.

### 2.2. Participants

For this study, we used data from a cross-sectional subsample of the children who participated in one of the two waves of data collection. We first included children who completed the mixed sentence repetition task during wave 2 (*n* = 56). We then added children who dropped out in wave 2 but did complete the mixed sentence repetition task during wave 1 (*n* = 4). This resulted in an initial dataset of 60 children. All children spoke English, Polish, or Turkish, either as the sole language at home or besides Dutch. Children who were not exposed to Dutch at home learned Dutch through daycare, school, and the local community. Study inclusion depended on several pre-registered criteria: (a) all children had to be between 4;0 and 7;11 years old at the time of participation; (b) children were exposed to both languages at least 15% of the time (i.e., either current or cumulative exposure; de Cat et al., 2025) or within the observed range of vocabulary scores as measured by the Cross-Linguistic Lexical Task (CLT; Haman et al., 2015); and (c) children were not exposed to a third language more than 15% of the time as measured by Q-BEx (de Cat et al., 2025). Some children were excluded because they (a) did not meet these inclusion criteria (*n* = 2); or (b) failed to comprehend the task (*n* = 1). Another group of children (*n* = 4) made more than four errors on each sentence. To assess whether inclusion of these children would affect the results, we ran our analyses both with and without these children. The results showed that the inclusion of these children did not affect the outcomes of interest or the associated conclusions (see OSF for Supplementary Analyses). Therefore, we present the results of our analyses with data from these children included.

The final sample included 57 children, consisting of English-Dutch (*n* = 11), Polish-Dutch (*n* = 15), and Turkish-Dutch (*n* = 31) children. All children were between 50 and 86 months old (*M* = 67.3, *SD* = 9.9 months) and 61.4% were girls. Demographic characteristics of the sample per language pair and word order group are summarized in Table 3. The three language pairs and two word order groups did not significantly differ in age, gender, parental education level, language exposure, and vocabulary knowledge in both languages. Turkish-Dutch children scored significantly lower on nonverbal reasoning than English-Dutch children (*F*(2, 54) = 3.336, *p* = 0.043). However, this difference was no longer significant when the English- and Polish-Dutch children were grouped together in the SVO sample (*F*(1, 55) = 2.743, *p* = 0.103), as is the case in our analyses.

### 2.3. Materials

#### 2.3.1. The Mixed Sentence Repetition Task

The mixed sentence repetition task was used to assess children’s ability to repeat mixed sentences. During the task, children are asked to repeat 20 sentences, either monolingual or mixed. All mixed sentences are in Dutch with switches to either English, Polish, or Turkish. All test sentences in the task are complex clauses that consist of a main clause and a subordinate clause that begins with a Dutch conjunction: ‘omdat’ because, ‘als’ if, ‘voordat’ before, ‘nadat’ after, ‘om’ so, ‘dat’ that, or ‘zodat’ such that. Research on Dutch first language acquisition has shown that children use their first subordinate clauses between 3 and 3.5 years of age (Wijnen & Verrips, 1998). This means that the sentence types in the task are appropriate for the age range tested (i.e., between 4 and 7 years old).

The mixed sentences include language mixing in either the main or the subordinate clause. Moreover, the task includes two types of mixing: alternation and insertion. These manipulations led to four types of mixed sentences (example sentences listed below): (1) insertion in the main clause; (2) insertion in the subordinate clause; (3) alternation in the main clause; and (4) alternation in the subordinate clause. In addition, the task includes monolingual Dutch sentences with similar structure but without switches to another language (see example 5 below). All test sentences are available on the OSF. To make sure that the overall number of sentences was doable for children aged 4–7 years in the context of a larger test battery, each condition was limited to four test sentences. All sentences are approximately the same length (i.e., between 8 and 13 words; there are no significant differences between languages or sentence types in sentence length) and contain words that are most likely familiar to children of this age (based on age of first acquisition in Dutch; Brysbaert et al., 2014).

(1) De jongen pakt de PLECAK als hij naar school gaat.
  “The boy grabs the BACKPACK when he goes to school.”
(2) De jongen pakt de blokjes om een EV te bouwen.
  “The boy grabs the blocks to build a HOUSE.”
(3) Het meisje PUSHES THE CHAIR zodat ze erlangs kan lopen.
  “The girl PUSHES THE CHAIR so she can walk past it.”
(4) De juf is boos omdat de kinderen ONU DINLEMIYOR.
  “The teacher is angry because the children DON’T WANT TO LISTEN TO HER.”
(5) De poes wil dat de kinderen haar aaien.
  “The cat wants the children to pet her.”

The sentences were constructed by native and highly proficient speakers of Dutch, English, Polish, and Turkish who were part of the research team, discussed within the full research team, and, based on discussion and agreement, adjusted if deemed necessary. The sentences were first constructed in Dutch in such a way that (1) translation to English, Polish, and Turkish was possible without large differences between the structures of the three language versions, and (2) sentence structure was similar across the three versions. This led to some deviations from the original Turkish switched parts, mostly in case of cognates (e.g., ‘appel’ apple, changed to ‘orange’ in the English-Dutch version). In case of insertion, the determiner was always in Dutch and only the noun itself was switched, in line with the MLF model (Myers-Scotton, 1993) and following other experimental mixing tasks (e.g., Byers-Heinlein et al., 2017). Furthermore, all nouns in Polish are male, because female nouns turned out to be hard to switch as, in Polish, female nouns should be inflected with some of the verbs used in the task. Male noun forms remain the same as in the nominative case, making it easier to implement grammatically correct switches.

Sentences were pre-recorded by (near-)native female speakers of both languages. Recordings were then integrated into a PowerPoint© presentation for test administration. The recorded sentences were presented using audio headphones. In case a child refused to wear the headphones, the task was presented through the speakers of the laptop. The task was administered by bilingual research assistants. Children were told that a woman on the computer was going to say something and that they had to repeat it verbatim. To familiarize children with the task, the task began with three practice sentences that included either an insertion (practice items 1 and 2) or alternation (practice item 3). During the first two practice items, the bilingual research assistant also participated (i.e., repeating the sentence and then asking the child to do the same) to encourage children to repeat the sentence. During the third practice item, the child was told that only they had to repeat the sentence. In case the child responded incorrectly during a practice item, the practice item was repeated. Children were told the research assistant would not participate anymore before starting the test items. All test items were administered in a fixed, pseudo-random order. When a child produced only one word (or nothing) for three consecutive test sentences, the research assistant aborted the task. Responses were recorded via an external USB microphone for subsequent offline scoring.

All sentences were transcribed and coded in Excel. Research assistants coded the total error rates (e.g., omissions, replacements) children produced per sentence, which was used as the outcome variable in this study. All assistants double-coded two participants (i.e., one Polish and one Turkish participant) to check reliable coding across assistants, resulting in good interrater reliability (kappa = 0.736). The coding protocol for the task can be found on the OSF.

#### 2.3.2. Cross-Linguistic Lexical Task (CLT)

The CLT (Haman et al., 2015) was used to measure vocabulary knowledge in both languages, which was in turn used to calculate language balance scores (i.e., relative language proficiency). Language balance was a covariate in the current study, based on earlier research using mixed sentence repetition tasks (Hofweber & Marinis, 2023; Soesman & Walters, 2021). The CLT includes four parts of 32 items each, i.e., (1) receptive knowledge of nouns, (2) receptive knowledge of verbs, (3) expressive knowledge of nouns, and (4) expressive knowledge of verbs. During the receptive parts, children were presented with four pictures and instructed to point to the picture that matched the word they heard. During the expressive parts, children were presented with one picture that they had to name. The order of the receptive and expressive parts was counterbalanced. The CLT is considered a valid measure of vocabulary knowledge (van Wonderen & Unsworth, 2021). The receptive and expressive scores were summed to create an overall vocabulary score per language (ranging from 0 to 128). From the overall vocabulary scores, we calculated balance scores by dividing the child’s score in the weaker language by the score in the stronger language (Tulloch & Hoff, 2023). This resulted in balance scores ranging from 0 to 1, with lower scores reflecting less balanced proficiency and higher scores reflecting more balanced proficiency.

#### 2.3.3. Quantifying Bilingual Experience Questionnaire (Q-BEx)

The parental Q-BEx questionnaire (de Cat et al., 2025) was used to measure mixing experience in daily life to investigate the ecological validity of the mixed sentence repetition task (Hypothesis 7). The questionnaire includes two fixed and several optional modules, one of which is about language mixing. This language mixing module includes questions about children’s exposure to and their own use of language mixing, including one-word and two-or-three-word switches. Parents were asked to indicate how often children are exposed to or use these mixing types on a 5-point Likert scale, ranging from “almost never” to “more than five times a day”. For ease of interpretation, we translated these scales into approximate frequency of mixing per week (with “almost never” corresponding to 0 times per week and “more than five times a day” corresponding to 42 times per week; Verhoeven et al., 2026; van Witteloostuijn & Blom, 2025). In order to compare children’s experience with intrasentential mixing in daily life with their performance on intrasentential mixing (i.e., insertion and alternation) in the mixed sentence repetition task, we included the frequency of mixing per week in Dutch on four questions: (1) exposure to one-word switches; (2) exposure to two-or-three-word switches; (3) production of one-word switches; and (4) production of two-or-three-word switches. One overall language mixing experience score was generated by summing the responses to these four questions, resulting in a maximum score of 168 (i.e., four questions × 42 times per week).

### 2.4. Analyses

To test Hypotheses 1–6, we constructed generalized linear mixed-effects models (GLMMs) using the glmer function from the lme4 package (version 1.1-37; Bates et al., 2015) for R software (version 4.5.0; R core team, 2024). The error rate per sentence in the mixed sentence repetition task was the outcome variable (i.e., count data). From the mixed sentence repetition task described above, we included three predictor variables, namely language condition (i.e., monolingual vs. mixed sentences), clause type (i.e., main vs. subordinate clause), and mixing type (i.e., insertion vs. alternation). Since linear mixed-effects models are well-versed at handling missing data at the item level (Baayen et al., 2008), we were able to include children who had missing data at the level of individual items. The model was constructed with categorical fixed effects and two continuous covariates (mean-centered age, language balance). We had preregistered to code the categorical predictors as orthogonal contrasts, but this proved unfeasible due to the overlap of the monolingual sentences across language condition, clause type and mixing type. To resolve this issue, we (a) included the categorical predictors in the model as factors that compared monolingual to mixed sentences, main clauses to subordinate clauses, and insertions to alternations; and (b) ran separate models: one for language condition (Hypothesis 1) on all sentences, and one for clause type, word order group and mixing type (Hypotheses 2–6) on mixed sentences only. In consultation with an Utrecht University statistician, we also fitted a single model including all predictors. As interpretation of this model was complex and estimates were similar, we report two separate models for ease of interpretation. The single model is available on the OSF.

To test Hypothesis 1, we analyzed the fixed effect of language condition, comparing error rates on monolingual versus mixed sentences to determine if children had greater difficulty with mixed sentences. To test Hypotheses 2–5, we examined the interaction between clause type and word order group to determine whether children found it more difficult to repeat sentences including mixing in clauses with non-overlapping word order than those with overlapping word order. Significant interaction effects were visualized using the ggplot2 package (version 4.0.0; Wickham, 2016). Tukey-corrected post hoc pairwise comparisons within each clause type (Hypotheses 2 and 3) and group (Hypotheses 4 and 5) were computed. More specifically, to test Hypothesis 2, we compared within the SVO group (English- and Polish-Dutch) error rates in subordinate versus main clauses. To test Hypothesis 3, the same comparison was made for the SVO group (Turkish-Dutch). To test Hypothesis 4 and 5, we compared error rates of the SVO and SOV groups on main and subordinate clauses, respectively. To test Hypothesis 6, we considered the fixed effect of mixing type and compared error rates on sentences with insertions versus alternations.

The initial models had Poisson distribution and included random intercepts and random slopes at both the child- and item-level to consider individual variability. As these models resulted in nonconvergence, we used the allFit function of the lme4 package and the optimx package (version 2025-4.9) to further simplify the models (Nash & Varadhan, 2011), but model convergence was only achieved after removing random slopes from the models. These models with Poisson distribution had overdispersion, so we ran the GLMMs again with a negative binomial distribution using the glmmTMB package (version 1.1.13; Brooks et al., 2017). The final models with negative binomial distribution and random intercepts are interpreted in our Results section.

To test Hypothesis 7, we calculated partial Spearman’s Rank correlations between children’s total error rates on the mixed sentence repetition task and the overall language mixing experience score in daily life. As in the previous analyses, these correlations are controlled for (mean-centered) age and language balance. For all analyses, significance of effects was determined through 95% Confidence Intervals (CI) and an alpha-level of 0.05.

## 3. Results

The descriptive statistics for each language pair are presented in Table 4. For mixed sentence repetition, we present error rates per sentence as well as the total number of errors on the task and the mixed and monolingual sentences specifically. Children’s mixing experience and language balance scores are also presented. Table 5 shows the error rates per sentence for each language condition, clause type, and mixing type. As we are interested in the interaction between clause type and group, we also present the mean and standard deviation for SVO and SOV groups separately. The descriptives show large standard deviations, suggesting considerable variation between subjects and items.

### 3.1. Hypotheses 1–6: Model Results

The parameter estimates of the generalized linear mixed-effects models with subject- and item-level random intercepts are presented in Table 6. Regarding the covariates included in both models, age emerged as a significant predictor, meaning that older children made fewer errors. Language balance was not significant, indicating that error rates did not vary as a function of balanced language proficiency. As regards our predictors of interest, language condition was not significantly related to the error rates per sentence; thus, the results did not show evidence for a difference in children’s error rates on monolingual and mixed sentences (contrary to Hypothesis 1), although the wide CI indicates uncertainty on whether there actually is a null effect or a possible moderate positive effect (as also suggested by Estimate = 0.247). Clause type showed a significant moderate negative effect, demonstrating that children had significantly higher error rates on main compared to subordinate clauses. The effect of word order group was not significant, showing that there was no evidence of a significant difference in overall error rates between English- and Polish-Dutch versus Turkish-Dutch children. The interaction between clause type and word order group was significant (Hypotheses 2–5). We unpack this interaction below. Mixing type was not significant, indicating that we found no evidence for a difference in the error rates on sentences with insertions and alternations (contrary to Hypothesis 6).

**Figure 1:**
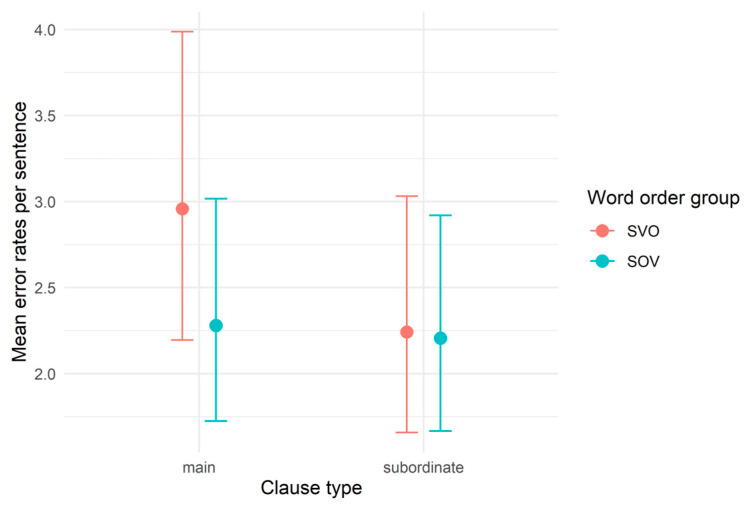
Mean error rates per clause type for each word order group.

To clarify the interaction between clause type and word order group, we ran Tukey-corrected post hoc pairwise comparisons of estimated marginal means (EMMs), which are visualized in Figure 1. First, we compared the error rates in main and subordinate clauses for the SVO and SOV groups separately (Hypotheses 2 and 3). The results indicated that the SVO group made significantly more errors on main compared to subordinate clauses (*z* = 2.065, *p* = 0.039), contrary to Hypothesis 2. Model-based EMMs showed an expected error rate of 2.96 (95% CI [2.20, 3.99]) on main clause mixed sentences compared to 2.24 (95% CI [1.66, 3.03]) on subordinate clause mixed sentences, with overlapping CIs indicating that the difference between clause types was small. For the SOV group, there was no significant difference (*z* = 0.254, *p* = 0.799), contrary to Hypothesis 3, with comparable error rates on main (*EMM* = 2.28, 95% CI [1.72, 3.02]) and subordinate clause mixed sentences (*EMM* = 2.21, 95% CI [1.67, 2.92]). Second, comparisons of the error rates between word order groups for each clause type (Hypotheses 4 and 5) revealed no significant difference on main (*z* = 1.434, *p* = 0.151; SVO: *EMM* = 2.96, 95% CI [2.20, 3.99]; SOV: *EMM* = 2.28, 95% CI [1.72, 3.02]) or subordinate clause mixed sentences (*z* = 0.089, *p* = 0.929; SVO: *EMM* = 2.24, 95% CI [1.66, 3.03]; SOV: *EMM* = 2.21, 95% CI [1.67, 2.92]), contrary to Hypotheses 4 and 5.

### 3.2. Hypothesis 7: Ecological Validity

To address the ecological validity of the mixed sentence repetition task, we calculated partial Spearman’s Rank correlations between the total error rates on the mixed sentences of the mixed sentence repetition task and the overall language mixing experience score in daily life. The correlation was very small and not significant (*r*(55) = 0.036, *p* = 0.794). Thus, in the current sample, there was no evidence of a relationship between the total error rates on the mixed sentences of the mixed sentence repetition task and mixing experience in daily life, contrary to Hypothesis 7.

## 4. Discussion

The present study examined how English-, Polish-, and Turkish-Dutch children between 4 and 7 years old processed and produced mixed sentences in a mixed sentence repetition task. We aimed to (1) study whether bilingual children have difficulty repeating sentences containing mixed language, (2) examine whether bilingual children’s ability to repeat mixed sentences is facilitated by shared word order, (3) explore the effects of mixing type (i.e., insertion and alternation) on bilingual children’s ability to repeat mixed sentences, and (4) study the relation between mixing experience in daily life and bilingual children’s ability to repeat mixed sentences. All in all, the results indicated no support for our hypotheses. We discuss these results and possible explanations for these findings below.

Regarding our first aim, we found no evidence for significant differences in error rates between mixed and monolingual sentences (Hypothesis 1), although error rates were numerically higher for mixed (*M* = 2.96, *SD* = 2.8) than for monolingual sentences (*M* = 2.52, *SD* = 3.0) and the wide 95% CI and Estimate suggest a possible moderate positive effect. Likewise, our results did not support the predictions for our second aim, as we found no indication that children had lower error rates when mixing occurred in the clause type that has word order overlap between languages compared to the clause type with no word order overlap (Hypotheses 2 and 3). In the SVO group, error rates were slightly higher on main clauses (with word order overlap) than on subordinate clauses (without overlap), though the effect was small, and overlapping CIs indicate that the effect should be interpreted with caution. Further, we found no evidence for differences between language pairs (Hypotheses 4 and 5). Thus, we found no evidence for a facilitating function of word order overlap in children’s language mixing. Concerning our third aim, we found no evidence for differences between insertions and alternations (Hypothesis 6), suggesting there are no additional processing and production costs associated with alternations compared to insertions. Finally, we found no evidence for a correlation between mixing in a sentence repetition task and mixing experience in daily life (Hypothesis 7), suggesting that children’s ability to repeat (mixed) sentences in the current task is not related to their naturalistic language mixing. However, this finding was based on a single correlation and warrants replication.

These findings are in not in line with previous research on language mixing and mixed sentence repetition specifically. While previous studies have suggested that the processing and production of mixed sentences is more costly than monolingual sentences (Gollan & Goldrick, 2016; Myers-Scotton & Jake, 2009; Soesman & Walters, 2021), this difference was not significant in the current study, contrary to Hypothesis 1. The lack of a significant difference in the current study may be a result of the relative ease with which bilingual children mix single nouns (Muysken, 2000), which made up 50% of the mixed sentences in the task. Moreover, theories on language mixing (Meisel, 1994; Myers-Scotton, 1993; Poplack, 1980) and observations based on naturalistic speech (Paradis et al., 2000; Yip & Matthews, 2016) suggest that word order overlap may facilitate mixing. However, our findings regarding word order overlap indicate no evidence of facilitation and may point to opposite effects for the SVO group, contrary to Hypotheses 2–5. Furthermore, our findings suggest that alternations may not require more grammatical integration than insertions (Muysken, 2000), contrary to Hypothesis 6. Finally, the lack of correlation between task performance and naturalistic language mixing are unlike previous studies that compared experimental tasks to mixing experience in daily life (Gross et al., 2022; Hofweber & Marinis, 2023). Taken together, the present findings provide no evidence for a role of language mixing in the sentence repetition performance of 4-to-7-year-old bilingual children, therefore offering no support for costs associated with processing mixed input and producing mixed output at this age and under these task conditions. This interpretation aligns with research reporting null to small relations between mixing in children’s input and their language outcomes (Place & Hoff, 2011, 2016; Verhoeven et al., 2026), similarly suggesting that language mixing may pose little difficulty for bilingual children. Of course, given the contrasting findings on mixed sentence repetition so far, these conclusions should be interpreted with caution and warrant replication in future studies using diverse methodologies and language pairs.

Beyond these theoretical considerations, methodological factors may also account for the null results observed in the present study. One methodological factor that may help to understand the null results regarding error rate differences in mixed sentence repetition, as well as the relation with mixing experience in daily life, concerns switch direction. Due to time restrictions, we included only switching from Dutch to the minority language. However, this direction may reflect children’s mixing in daily life less well than switching from the minority language to Dutch. A recent systematic review shows that children generally mix more from the minority to the majority language (Snijders et al., 2026), both in daily life (e.g., Meng & Miyamoto, 2012) and in structured tasks (e.g., Montanari et al., 2019). The inclusion of switches from the minority language to Dutch in our sentence repetition task would have been more natural, and therefore more suitable to detect subtle differences in processing and production costs and/or a relation with children’s mixing experience in daily life. In comparison, Soesman and Walters (2021) did include both switching directions. Their results showed that switching direction matters especially when there is less grammatical overlap. Moreover, the two languages included in their study (i.e., Hebrew and English) are more comparable in language status within the context they studied, compared to the language pairs in our study, specifically Polish-Dutch and Turkish-Dutch. As children are sensitive to the higher status of the majority language in society (Treffers-Daller, 2019) and mix more from the minority language into the majority language (Snijders et al., 2026), switching direction differences may be amplified in the current sample, such that effects of word order overlap, clause and mixing type were not found because of the switching direction from Dutch to the minority language. Therefore, future research should take into account both switching directions in relatively homogeneous samples of bilingual children who speak languages of unequal status.

Another methodological factor that may help explain the null results may be the use of accuracy as an indication of the processing (and production) costs associated with mixed sentence repetition, even though the other studies also used accuracy. Studies on mixed sentence processing in children found increased processing costs associated with mixed compared to monolingual sentences when using sensitive online measures such as proportional looking time (Byers-Heinlein et al., 2017), pupil size (Byers-Heinlein et al., 2017), or reaction times (RTs; Gross et al., 2019). In contrast, measures of offline comprehension, such as accuracy, do not always show differences between mixed and monolingual sentences (Gross et al., 2019), indicating that differences in processing costs may not always be visible in offline behavioral measures. Importantly, the absence of evidence for behavioral differences (accuracy, RTs) does not mean that processing is identical. More subtle, lower-level differences, such as neural or implicit processing differences, may still occur, as indicated by brain imaging research of sentence processing in adult bilinguals (Kovelman et al., 2008). It is thus conceivable that the measure of accuracy in our study was not sensitive enough to detect differences in additional processing costs associated with repeating different types of mixed sentences.

Moreover, sample size was relatively small in our study (*N* = 57), especially when considering the sample size per language group (English-Dutch, *n* = 11; Polish-Dutch, *n* = 15, and Turkish-Dutch, *n* = 31). To increase sample size, we collapsed our analyses across the English- and Polish-Dutch groups (i.e., SVO word order), yet English and Polish differ regarding other language characteristics (e.g., the use of articles, language status), which could potentially increase within-group variation and lower the likelihood of finding between-group differences. The same consequences may hold for between-language differences regarding, for example, grammar, syntax or noun choice in the test sentences. Importantly, the current study is among the first to study grammatical constraints in an experimental, controlled setting instead of through naturalistic case studies. At the same time, the various methodological limitations of the current study, including the use of only one switch direction, error rate as the outcome variable, the relatively small sample size, and differences between languages (e.g., language status, grammatical differences), warrant caution and highlight the need for further research and replication.

To better understand our findings in relation to other research, we delve more deeply into comparisons with the work of Soesman and Walters (2021), because this is the only previous study that used mixed sentence repetition with children. Unlike our study, they found differences in children’s accuracy between various sentence types. Several important methodological differences may help us interpret the differing results. First, while both studies target accuracy, the operationalization of the outcome measure differs. While we used error rate per sentence in the current study, Soesman and Walters (2021) used the proportion of sentences repeated verbatim per sentence type. The latter measure is based on a binary outcome of correct versus incorrect repetition on the sentence level. Binary coding of children’s responses can introduce artificially sharp boundaries, particularly for children near the cut-off (Rose et al., 2025). At the same time, it offers practical advantages in terms of data interpretation and statistical power. Still, potential pitfalls, such as amplified differences or distorted effect sizes (Altman & Royston, 2006) should be addressed by, for example, comparing binary analyses with continuous analyses. Second, the example sentence presented in Soesman and Walters (2021) is slightly shorter than the average sentence used in the current study. As children have more difficulties once sentences become longer—as sentence repetition tasks target grammatical knowledge in sufficiently long sentences (Klem et al., 2015)—our task may have targeted grammatical knowledge rather than processing (and production) efforts, which may have resulted in higher overall error rates in the current study. These higher overall error rates may have obscured more subtle differences in processing or production costs that would be detectable in tasks with shorter sentences, such as the ones in Soesman and Walters (2021). Finally, the chosen language pairs differ between studies. We chose three language pairs (i.e., English-, Polish-, and Turkish-Dutch) to capture effects of shared word order that differ across clause types. Soesman and Walters (2021) chose a language pair (i.e., English-Hebrew) that shares word order within prepositional phrases but differs in the theorized constraints on specific switch sites, like the free morpheme constraint (Poplack, 1980). This choice results in a focus on specific morphosyntactic phenomena, different from the focus on general word order in our study. Importantly, although their results partially support the hypothesized constraints, mixing in spontaneous speech does not always follow such constraints (Azuma, 1996). This indicates that further research is needed, both regarding specific morphosyntactic phenomena and more general word order differences as well as the link between mixing in a task and spontaneous speech.

An unexpected finding, not specified in our research questions or hypotheses, was that children made more errors in mixed main clauses than in mixed subordinate clauses, an effect that was stronger in the SVO group. This finding may be explained by known effects of serial sentence position on sentence processing and production. In various types of immediate recall experiments, participants often best recall initial information (primacy effect) or final information (recency effect) (e.g., Yoo & Kaushanskaya, 2016). Previous research on non-mixed sentence repetition indeed shows that children often have higher error rates in medial sentence position compared to initial and final position (Alloway & Gathercole, 2005; Stadtmiller et al., 2022), and these higher error rates are related to children’s lower memory capacities in medial but not initial and final position (Stadtmiller et al., 2022). Although this explanation is post hoc and error position analyses were beyond the scope of the current study, all mixed sentences included in the task were structured such that initial sentence position was always in Dutch, while mixes occurred either in a medial (i.e., in the main clause) or in the final position (i.e., in the subordinate clause). Mixing in the final position may have served as a saliency cue, which may have resulted in better recall of those sentences compared to sentences in which mixing occurred in the medial position. Furthermore, as mixing in the subordinate clause does not adhere to grammatical constraints for the SVO group, such mixing may draw extra attention and enhance monitoring efforts (Green & Abutalebi, 2013). As a result, these sentences may have been more salient in memory. In turn, children in the SVO group may have generally made more errors on mixed main clauses compared to mixed subordinate clauses, regardless of word order overlap. In comparison, mixed sentences in Soesman and Walters (2021) included a temporal phrase after the mixed prepositional phrases. Therefore, all mixing occurred in the medial sentence position and recency effects likely did not play a role in their study. It thus remains unclear how including mixes in different sentence positions affects error rates in a mixed sentence repetition task.

## 5. Conclusions

The current study is one of the first to address the role of word order overlap in language mixing in an experimental manner using a mixed sentence repetition task in a larger sample across three different groups of bilingual children in the Netherlands. We found no evidence that shared word order facilitates language mixing. Moreover, we found no evidence of differences in the processing costs regarding monolingual versus bilingual sentences or insertions versus alternations. Finally, we found no evidence of a relation between task performance and mixing experience in daily life. These results are not in line with several theories on language mixing (Gollan & Goldrick, 2016; Meisel, 1994; Myers-Scotton, 1993; Myers-Scotton & Jake, 2009; Muysken, 2000; Poplack, 1980) and may suggest that mixing plays a limited role in the costs associated with processing and production efforts. Moreover, the results might also reflect the ease with which bilingual children switch single nouns (Muysken, 2000), as about half of the sentences in this study included noun switches. Importantly, the study has some methodological constraints that limit our ability to draw conclusions and highlight the need for replication. These include sample size, outcome measure, and task characteristics such as switching direction and differences between languages (e.g., language status, grammatical differences). We provide suggestions for future studies to tackle these limitations when investigating children’s language mixing in mixed sentence repetition tasks, and in studying language mixing more generally. Despite these limitations, the current study provides initial evidence pertaining to the role of grammatical constraints in children’s language mixing. A better understanding of this role can clarify how children use their grammatical knowledge while mixing languages, which can be used to inform parents and language practitioners and ease their concerns regarding language mixing.

## Figures and Tables

**Table 1:** Word order, language mixing examples, and congruency of language mixing with word orders of the languages.

1a	Word Order Examples	1b	Language Mixing Examples	Congruent with	
i. Dutch SVO in main clause	Jan leest een boek John reads a book	v. Mixing in main clause		Dutch	Minority
		NL–EN	Jan leest een BOOK	Yes	Yes
		NL–PL	Jan leest een KSIAZKE	Yes	Yes
		NL–TR	Jan leest een KITAP	Yes	No
ii. Dutch SOV in subordinate clause	Marie ziet dat Jan een boek leest Mary sees that John a book reads	vi. Mixing in subordinate clause			
		NL–EN	Marie ziet dat Jan READS A BOOK	No	Yes
		NL–PL	Marie ziet dat Jan CZYTA KSIAZKE	No	Yes
		NL–TR	Marie ziet dat Jan BIR KITAP OKUYOR	Yes	Yes
iii. Polish and English SVO	Jan czyta ksiazke John reads (a) book				
iv. Turkish SOV	Jan bir kitap okuyor John a book reads				

Note. Green indicates less difficulty. Red indicates increased difficulty. We are aware that the English examples shown here include a Dutch-English cognate (i.e., boek–book). The mixed sentence repetition task that we administered did not include any cognates.

**Table 2:** Hypotheses regarding word order (Hypotheses 2–5).

	Main Clause	Subordinate Clause	
English			(2)
Polish			(2)
Turkish			(3)
	(4)	(5)	

Note. Green indicates less difficulty. Red indicates increased difficulty.

**Table 3:** Demographic characteristics of the sample per group.

	English-Dutch *M* (*SD*)	Polish-Dutch *M* (*SD*)	SVO *M* (*SD*)	SOV Turkish-Dutch *M* (*SD*)
*n*	11	15	26	31
Gender (% girls)	81.8	60.0	69.2	54.8
Age in months	65.8 (9.0)	67.3 (11.5)	66.7 (10.5)	67.9 (9.2)
Exposure %				
Dutch	51.9 (20.4)	54.2 (20.9)	53.2 (20.7)	54.3 (21.7)
Minority	48.1 (20.4)	44.8 (21.0)	46.2 (20.8)	44.9 (21.6)
L3 (if applicable)	NA ^c^	3.8 (1.1)	3.8 (1.1)	8.7 (4.1)
Parent education level % ^a^				
Secondary school	0	0	0	6.5
Post-Secondary	9.1	13.3	22.5	12.9
University degree	90.9	86.7	88.5	80.6
Nonverbal reasoning	11.4 (3.5)	9.1 (2.4)	10.1 (3.1)	8.8 (2.8)
Vocabulary Dutch ^b^	94.8 (12.1)	86.9 (16.5)	90.2 (15.1)	90.2 (15.5)
Vocabulary minority ^b^	102 (10.5)	90.5 (22.9)	95.3 (19.3)	83.7 (25.8)

^a^ Parent education level was measured as the highest level of education attained by either parent via the Q-BEx questionnaire (de Cat et al., 2025). ^b^ Vocabulary in both languages is presented as the sum of raw scores on comprehension and production of both nouns and verbs of the CLT (Haman et al., 2015). ^c^ NA = Not Applicable.

**Table 4:** Descriptive statistics per language pair.

	SVO						SOV		
	English-Dutch (*n* = 11)			Polish-Dutch (*n* = 15)			Turkish-Dutch (*n* = 31)		
	*M*	*SD*	Range	*M*	*SD*	Range	*M*	*SD*	Range
Error rates									
Per sentence	2.32	2.3	0–16	3.18	2.8	0–12	2.95	3.0	0–15
Total task	46.46	26.3	7–109	56.93	24.4	22–106	58.52	43.3	9–186
Total mixed	38.63	21.9	7–90	46.53	20.4	16–91	48.39	34.6	9–146
Total mono	7.82	5.4	0–19	10.40	7.2	1–23	10.13	9.5	0–40
Mixing experience	56.96	57.8	1.5–168	45.50	36.8	0–94.5	44.71	46.0	0–168
Language balance	0.91	0.1	0.80–0.98	0.83	0.1	0.63–0.99	0.73	0.2	0.39–0.98

**Table 5:** Error rates per sentence for model variables.

	Full Sample (*N* = 57)		SVO (*n* = 26)		SOV (*n* = 31)	
	*M*	*SD*	*M*	*SD*	*M*	*SD*
Language condition						
Monolingual	2.52	3.0				
Mixed	2.96	2.8				
Clause type						
Main clause	3.18	2.92	3.24	2.5	3.13	3.2
Subordinate clause	2.75	2.68	2.51	2.6	2.94	2.71
Mixing type						
Insertion	2.85	2.8				
Alternation	3.08	2.7				

**Table 6:** GLMMs predicting the error rates per sentence.

	Estimate	95% CI	SE	*z*	*p*
Model Hypothesis 1					
Language condition	0.247	[−0.111, 0.606]	0.183	1.351	0.177
Age	−0.313	[−0.478, −0.148]	0.084	−3.710	<0.001 *
Language balance	−0.107	[−0.271, 0.056]	0.084	−1.286	0.198
Model Hypotheses 2–6					
Clause type	−0.277	[−0.540, −0.014]	0.134	−2.065	0.039 *
Word order group	−0.260	[−0.616, 0.095]	0.182	−1.434	0.151
Clause type * group	0.244	[0.060, 0.428]	0.094	2.596	0.009 *
Mixing type	0.113	[−0.128, 0.355]	0.123	0.919	0.358
Age	−0.286	[−0.447, −0.126]	0.082	−3.494	<0.001 *
Language balance	−0.136	[−0.308, 0.037]	0.088	−1.543	0.123

Note. * significant at *p* = 0.05 level.

## Data Availability

The data used in the current study are publicly available on the Open Science Framework at: https://osf.io/96z8d. The raw data behind the dataset are not available as the study is part of an ongoing research project. Requests to access the raw data should be sent to the corresponding authors.

## Note

1 In the literature, different terms (e.g., code-switching, code-mixing or language mixing) are used to refer to the same phenomenon (see Montanari et al., 2019 for an elaborate discussion). The term language mixing is used in this study to describe the alternation of languages, which is the common terminology used in child research (Meisel, 1994).

## Supplementary Materials

The following supporting information can be downloaded at: https://osf.io/96z8d, mixed sentence repetition task test sentences, coding protocol, data analysis scripts, and supplementary analyses.
